# The Tobacco Industry and Pesticide Regulations: Case Studies from Tobacco Industry Archives

**DOI:** 10.1289/ehp.7452

**Published:** 2005-08-08

**Authors:** Patricia A. McDaniel, Gina Solomon, Ruth E. Malone

**Affiliations:** 1 Center for Tobacco Control Research and Education, University of California, San Francisco, California, USA; 2 Natural Resources Defense Council, San Francisco, California, USA; 3 Division of Occupational and Environmental Medicine, Department of Medicine, University of California, San Francisco, California, USA; 4 Department of Social and Behavioral Sciences and School of Nursing, University of California, San Francisco, California, USA

**Keywords:** ethylene bisdithiocarbamates, Environmental Protection Agency, methoprene, pesticide regulation, phosphine, tobacco industry, World Health Organization

## Abstract

Tobacco is a heavily pesticide-dependent crop. Because pesticides involve human safety and health issues, they are regulated nationally and internationally; however, little is known about how tobacco companies respond to regulatory pressures regarding pesticides. In this study we analyzed internal tobacco industry documents to describe industry activities aimed at influencing pesticide regulations. We used a case study approach based on examination of approximately 2,000 internal company documents and 3,885 pages of U.S. Environmental Protection Agency documents obtained through Freedom of Information Act requests. The cases involved methoprene, the ethylene bisdithiocarbamates, and phosphine. We show how the tobacco industry successfully altered the outcome in two cases by hiring ex-agency scientists to write reports favorable to industry positions regarding pesticide regulations for national (U.S. Environmental Protection Agency) and international (World Health Organization) regulatory bodies. We also show how the industry worked to forestall tobacco pesticide regulation by attempting to self-regulate in Europe, and how Philip Morris encouraged a pesticide manufacturer to apply for higher tolerance levels in Malaysia and Europe while keeping tobacco industry interest a secret from government regulators. This study suggests that the tobacco industry is able to exert considerable influence over the pesticide regulatory process and that increased scrutiny of this process and protection of the public interest in pesticide regulation may be warranted.

Tobacco is a pesticide-intensive crop. With nearly 27 million pounds of pesticides (including insecticides, herbicides, fungicides, and suckercides) applied to the U.S.-grown crop from 1994 to 1998, it ranks sixth in terms of the amount of pesticides applied per acre [U.S. Government Accounting Office (GAO) 2003]. The tobacco industry regards pesticides as essential to tobacco production, stating that “the crop could not be produced economically without them” (Davis 1989; Philip Morris 1990b). According to industry documents, government-imposed limitations on pesticide use “may present a serious impediment” to the international tobacco trade (Hill 1989).

Internal tobacco industry documents provide a window into the tobacco industry’s activities regarding pesticide regulations. These case studies drawn from industry documents describe the tobacco industry’s responses to pesticide regulatory action. The documents also provide insight into the relationships between the tobacco industry and pesticide regulatory agencies and tensions between business and public health interests.

## The Tobacco Industry Documents

Litigation against the tobacco industry has resulted in the release of nearly 7 million previously secret tobacco industry documents (Bero 2003; Malone and Balbach 2000). Scanned PDF versions of original handwritten, typed, or printed documents have been archived at the University of California, San Francisco, library in electronic repositories, searchable using basic keywords (http://legacy.library.ucsf.edu). Between July 2003 and February 2004, we searched the archives using a “snowball” sampling strategy, beginning with broad search terms (“pesticide” and “crop protection agent”) and using retrieved documents to identify more specific search terms (such as names of specific pesticides, people, and regulatory agencies). Table 1 provides examples of keyword searches and the number of documents yielded. This process produced nearly 300,000 documents relating to many different pesticides. The first author reviewed these documents’ index entries and excluded duplicates and documents unrelated to pesticide regulatory issues. The final sample size was approximately 2,000 documents, spanning 1974–2001.

We also filed Freedom of Information Act (FOIA) requests with the U.S. Environmental Protection Agency (EPA) on pesticide issues raised by information in the industry documents, resulting in 3,885 pages of government documents. Finally, we reviewed public health agency reports based on industry documents (Zeltner et al. 2000). We analyzed the industry, government, and public health agency documents by assembling chronologically constructed case studies, a method common to sociology, political science, and anthropology (e.g., analyses of a corporation’s organizational structure, a social movement, or a tribe) (Hill 1993; Yin 1994) (Table 2). The pesticides chosen for inclusion [methoprene, the ethylene bisdithiocarbamates (EBDCs), and phosphine] were those for which sufficient information related to regulatory activities was available in the archives to construct a case study.

## Pesticides and Tobacco

Pesticides used on tobacco are also used regularly on food crops. As with food crops, trace amounts of pesticides remain on tobacco leaves after treatment; typically, residue levels decline during the drying and manufacturing process, although additional pesticides may be applied to the finished product (U.S. GAO 2003). Although pesticides increase production of tobacco and food crops, pesticide exposure may harm humans; thus, regulatory agencies such as the U.S. EPA may set limits on the amount of pesticide residue permitted in or on food and tobacco and establish standards for workers handling pesticides. Because tobacco is burned and the smoke inhaled, active and passive smokers are exposed to pyrolyzed pesticide residues (U.S. GAO 2003). The U.S. EPA has concluded that this exposure poses no short-term risk, but little is known about the long-term health effects (U.S. GAO 2003).

## Methoprene

In 1974, Philip Morris formed a partnership with the chemical company Zoecon to market a new insecticide (Manzelli 1975). The insecticide’s active ingredient, methoprene, acted as an endocrine disruptor in cigarette beetles and tobacco moths, preventing their larvae from maturing into adult insects (Manzelli 1975). Philip Morris anticipated that methoprene would replace phosphine, a common warehouse fumigant (Philip Morris 1988) and pledged to assist Zoecon in introducing methoprene “in as many countries as we can” (Seligman 1982).

Some countries have regulations that require the establishment of maximum residue limits (MRLs) for pesticides on crops; however, Philip Morris determined that MRLs were not required in all countries, especially for pesticides on nonfood crops such as tobacco (Ryan 1991). Philip Morris asked Zoecon “to not force this issue and submit for MRLs when not required” (Lindahl 1992b). In April 1991 Zoecon alerted Philip Morris’s director of research that the Malaysian pesticide board had recently set an MRL of 1.0 ppm for methoprene on tobacco (Hutney 1991). Zoecon considered 1.0 ppm too low to enable the effective use of methoprene; the level supported by the labeled application rate was 10 ppm (Ryan 1992). Philip Morris requested that Zoecon ask for an even higher MRL of 15 ppm to allow for application errors (Greenberg and Transon 1992; McCuen 1992). Zoecon representatives met with government authorities and requested a change to 15 ppm (Hutney 1991).

A Zoecon representative informed Philip Morris that “in order to avoid surprises of this nature in the future,” he had directed Zoecon’s pharmaceutical group to obtain information from health authorities in other countries regarding the commodities for which methoprene tolerances were assigned (which could include foods such as rice and mushrooms as well as tobacco) (Hutney 1991). Assigning this task to the pharmaceutical group instead of the pesticide group, the Zoecon representative wrote, “will not arouse the curiosity of the health directorates and will allow us to keep our promise to the tobacco industry, namely, that we won’t initiate queries that may cause the health authorities to direct attention to tobacco” (Hutney 1991).

In April 1992, George Lindahl of Zoecon faxed a letter to Bob McCuen, head of Philip Morris’s biochemical research, outlining some of his concerns about Philip Morris’s approach to establishing MRLs for methoprene on tobacco (Lindahl 1992b). In regard to Zoecon’s effort to establish an MRL of 15 ppm in Malaysia, Lindahl explained that

I know we simply argued this case without any data to support our request. In more advanced countries, this tactic will not succeed. … All our data demonstrate the need for a 10 ppm MRL. If a higher value is desired then we will require data from real field operations showing that a worse [*sic*] case scenario for faulty application will result in a 15 ppm residue, and hence the need for this value. (Lindahl 1992b)

In a fax following this one, Lindahl asked Philip Morris to provide such data; a handwritten comment from a Philip Morris employee who reviewed the fax noted that “data doesn’t [*sic*] exist” (Lindahl 1992a). Initially, the Malaysian authorities agreed to increase methoprene’s MRL to 10 ppm (Lindahl 1992c); subsequently, it was raised to 15 ppm (Mueller and Ward 1998). Philip Morris continued to advocate (through Zoecon) for MRLs of 15 ppm in Italy and Germany (Greenberg and Transon 1992).

In the meantime, anticipating the creation of a single European market with uniform pesticide regulations, Philip Morris had asked the longtime tobacco industry law firm, Shook, Hardy, and Bacon, to prepare a document with MRL recommendations for possible submission to the European Community (Kemna 1991). Philip Morris first provided a draft of recommended MRLs to the Scientific Working Group of the Confederation of European Community Cigarette Manufacturers (CECCM) (Philip Morris 1991c). At their June 1991 meeting, members of this group (including representatives of Philip Morris, British American Tobacco, R.J. Reynolds, Gallaher, and Rothmans) recommended that the document be rewritten as a voluntary code of practice “to be used pre-emptively … in advance of any EC [European Community] initiative” to impose formal regulations on pesticide residue limits on tobacco (Philip Morris 1991a). A meeting participant reported, “It is hoped that, by implementing this Code, the EC Commission would not any longer see the need to develop a formal EC regulation on pesticide residues in tobacco (products)” (Mueller 1991). Manuel Bourlas, Philip Morris’s director of research and development, was appointed chair of a subgroup of tobacco company representatives who were to assist in preparing the code (Philip Morris 1991a).

This voluntary code underwent numerous revisions throughout 1991 and 1992 (CECCM 1991, 1992a, 1992b, 1992c, 1992d, 1992e; Philip Morris 1991b). Although 236 regulated and unregulated tobacco pesticides were in use at the time (Mitchell 1991b), the voluntary code proposed MRLs for only 25–27 pesticides [including chlordane, dichlorodiphenyltrichloroethane (DDT), lindane, dithiocarbamates, methoprene, and maleic hydrazide]. According to British American Tobacco’s Terry Mitchell, “many of the substances in the list are no longer recommended for tobacco production” (e.g., DDT) (Mitchell 1991a). Moreover, this list did not impose “any constraint automatically on non-specified substances” (Mitchell 1991a). Mitchell noted that this lack of limits was “highly desirable” (Mitchell 1991a).

In December 1992, Walter Russell, a legal assistant, reported that the code “has undergone two more revisions (by SHB) [Shook, Hardy, and Bacon] and it [is] currently watered down, but still causing much agitation” (Philip Morris 1992). Russell pointed out that the code set MRLs that Philip Morris “might have trouble complying with” if they were to become international standards (Philip Morris 1992). In addition, “failure to comply with tolerances written by the tobacco industry which might come up during litigation would put the tobacco industry at great disadvantage” (Philip Morris 1992). He indicated that Philip Morris had decided to withdraw its support from the voluntary code (Philip Morris 1992). In 1993, the tobacco companies suspended work on the document due to “principle disagreements both within and between participating companies” (R.J. Reynolds 1993). Throughout the 1990s, the tobacco industry continued to anticipate European Union harmonization of tobacco pesticide MRLs (Philip Morris 1995); as of April 2004, the European Union had established community-level MRLS for 150 pesticides, but none specifically applied to tobacco (European Union 2004).

## EBDC Fungicides

In 1987, the U.S. EPA initiated a review of EBDC fungicides, prompted by the agency’s determination that a breakdown product of EBDCs, ethylene thiourea (ETU), was a probable human carcinogen (U.S. EPA 1987). Anticipating the U.S. EPA’s cancellation of many EBDC uses, U.S. manufacturers voluntarily withdrew EBDC registrations for all but 13 food crops in 1989, including wheat and corn (U.S. EPA 1989). At least one company continued to hold registrations for EBDCs on tobacco, but only for seed bed use, not plants (Arce 1989).

In internal documents, the tobacco industry expressed concern that the U.S. EPA’s action could result in the “imposition of potentially crippling product residue tolerances” in Europe [Centre de Coopération pour les Recherches Scientifiques Relatives au Tabac (CORESTA) 1989b; Mitchell 1990]. EBDCs were regarded as vital to control blue mold outbreaks in Europe (Philip Morris 1990a). In October 1989, members of CORESTA, an international tobacco research organization with members drawn largely from the tobacco industry, established a subcommittee to “provide regulatory agencies with a sound basis for the development of tobacco agro-chemical regulations” (CORESTA 1989a, 1989b).

As discussed in a larger World Health Organization (WHO) report on tobacco industry influence at that agency, the subcommittee hired a consultant, Gaston Vettorazzi, to provide advice on influencing regulation (CORESTA 1990b; Zeltner et al. 2000). Vettorazzi was a former WHO toxicologist and former technical secretary of the Joint Food and Agriculture Organization/WHO Meeting on Pesticide Residues (JMPR), an international meeting of scientists whose decisions often formed the basis of international law (Zeltner et al. 2000). Selected partly for his “old boys’ contacts” (Reif 1991b), Vettorazzi’s initial duties were to provide a review and analysis of toxicologic data on EBDCs and ETU (CORESTA 1990a).

Some CORESTA members were concerned that Vettorazzi’s review might conclude that EBDCs were unsafe (Beuchat 1990). However, according to one member’s notes, at his first meeting with the subcommittee in April 1990, Vettorazzi stated that “someone has to lay the red carpet for [me], otherwise [I] can spoil more than help” (Reif 1990).

Vettorazzi’s initial review concluded that ETU was neither carcinogenic nor genotoxic (Vettorazzi 1991a). Some of the tobacco industry scientists commented that this statement was “too strong in light of the NTP feeding studies”—a reference to the U.S. National Toxicology Program’s conclusion that animal studies showed clear evidence of ETU’s carcinogenicity (Reif 1991a). Vettorazzi subsequently revised his conclusions, stating that ETU’s “toxicity, including carcinogenicity, can be explained by the known mechanisms of action characteristic of thyroid-function inhibiting agents” (Vettorazzi 1991b). Thus, he stated, a threshold could be set below which ETU did not cause thyroid tumors (Vettorazzi 1991b).

CORESTA authorized the distribution of Vettorazzi’s revised report to his former colleagues at WHO, once all references to tobacco and CORESTA were removed (CORESTA 1992). WHO’s JMPR was scheduled to review EBDCs/ETU in 1993; if this review were favorable, the tobacco industry would be assured continued access to EBDCs in Europe (Zeltner et al. 2000).

With CORESTA funding ($100,000 a year) and approval, Vettorazzi offered to assist J. Herrman, of the JMPR WHO Secretariat, with JMPR toxicologic reviews, without disclosing his tobacco industry ties (Herrman 1991; Vettorazzi 1991c, 1992a). Vettorazzi wrote and reviewed several working papers on compounds to be discussed at the 1992 JMPR, including the EBDC thiram (Herrman 1992; Vettorazzi 1992b). One outcome of that meeting was the reestablishment, at a higher level, of the previously cancelled Acceptable Daily Intake (ADI) for thiram (Vettorazzi 1992b).

Vettorazzi continued his work with WHO in 1993, supplying his CORESTA-funded reviews to the adviser responsible for drafting the working paper that would form the basis of the September JMPR on EBDCs/ETU without revealing their sponsor (Zeltner et al. 2000). Vettorazzi also attended the September meeting as an invited “temporary adviser” (Zeltner et al. 2000). The meeting’s outcome reflected Vettorazzi’s conclusions. In contrast to the U.S. EPA, JMPR determined that ETU was not genotoxic, and thus raised the ADI level from 0.002 to 0.004 mg/kg body weight (Black 1993). CORESTA considered this “a very positive result for the industry,” since it “clearly indicates that the ‘carcinogenicity’ of [ETU] is not really a burning issue any longer” (CORESTA 1994; Mueller 1993). JMPR’s safety standard became part of international trade law, preserving tobacco industry access to EBDCs (Zeltner et al. 2000). Soon after the JMPR meeting, CORESTA extended Vettorazzi’s contract for 18 months, listing one of his duties as providing “information about the activities of pesticide action groups” (CORESTA 1993). He was to be paid another $100,000 (CORESTA 1993). Vettorazzi continued working for CORESTA until at least 2001, when the organization paid him $30,000 to monitor international activities related to tobacco pesticide residues and registrations (CORESTA 2001).

## Phosphine

Phosphine is a fumigant used on stored commodities, including nuts, seeds, grains, coffee, tobacco, and finished cigarettes to kill insects. Because of the risks it poses, applicators are advised to wear respirators and protective clothing, and warehouses must be sealed to prevent leaks that contribute to air pollution and endanger nearby residents (U.S. EPA 1998b). By the early 1990s, several case reports had been published noting sometimes fatal phosphine poisoning among workers and community members (Garry et al. 1989, 1993; Heyndrickx et al. 1976; Schoonbroodt et al. 1992; Wilson et al. 1980).

In December 1998, the U.S. EPA proposed a series of 15 risk mitigation measures (RMMs) for phosphine. The U.S. EPA’s primary concern was the risk that phosphine posed to applicators and community residents (U.S. EPA 1998b). Thus, the RMMs included a threshold limit value of 0.03 ppm of phosphine during fumigation (reduced from the existing 0.3-ppm standard), the establishment of a 500-foot buffer zone around all fumigated structures, and prior notification of all residents living within 750 feet of a fumigated structure (U.S. EPA 1998a).

The Tobacco Association of the United States, in a letter to the U.S. EPA, stated that the economic burdens imposed by the RMMs would “make it virtually impossible for our industry to continue to fumigate stored tobacco” (Ward 1999). The Tobacco Association, R.J. Reynolds, Philip Morris, and > 150 other organizations with a stake in the continued use of phosphine formed a lobbying group, the Commodity Industry Coalition for Phosphine Fumigation (Harrell 1999).

R.J. Reynolds, represented primarily by toxicologist Joel Seckar, took an active role in the Commodity Industry Coalition (Seckar 1999c). The company calculated that complying with the U.S. EPA’s buffer zone requirement would cost approximately $50 million in new land and warehouse purchases (R.J. Reynolds 1999a). Increasing the time required to aerate warehouses before employee reentry to comply with the worker exposure limit of 0.03 ppm would increase costs, as would the possibility of liability suits brought by nearby residents notified of phosphine use (Degesch America 1998; R.J. Reynolds 1999d).

Coalition members lobbied Congress, released media statements, worked closely with the U.S. Department of Agriculture, and attended U.S. EPA-sponsored stakeholder meetings (Goldman 1998; Lyon 1999; R.J. Reynolds 1999b, 1999c). Their message was that the proposed RMMs were overly conservative, based on “anecdotal information and hypothetical risk” rather than on “sound science” (Lyon 1999; Ong and Glantz 2001). To challenge the scientific basis of the U.S. EPA’s proposals, the coalition decided to hire an expert whose research would support existing standards (Seckar 1999h). They chose Sciences International, a consulting firm specializing in health and environmental risk assessment. It was headed by Elizabeth Anderson, a former director of the Carcinogen Assessment Group and the Office of Health and Environmental Assessment at the U.S. EPA (Sciences International 2005). She was also an experienced expert defense witness, having served in that capacity in a number of environmental lawsuits brought against corporations (Anderson 1999c).

To support the Commodity Industry Coalition’s assertion that the proposed exposure level of 0.03 ppm was too conservative, Sciences International focused on the inter-species uncertainty factor. The U.S. EPA had first determined from a published subchronic toxicity study of rats that there were no observed effects attributable to inhaled phosphine at 3 ppm (Seckar 1999a). To extrapolate to humans, the U.S. EPA had then used two 10-fold uncertainty factors, one for intraspecies variability and one for interspecies variability, to arrive at a maximum exposure level of 0.03 ppm (Sciences International 1999c). Documents indicate that Sciences International’s strategy was to convince the U.S. EPA that the interspecies uncertainty factor was unnecessary, showing that because a number of animal species reacted in the same manner to phosphine, humans were similar enough that the interspecies uncertainty factor could be removed (Seckar 1999a, 1999b). This would leave only the intraspecies factor of 10, which would result in a maximum exposure level for humans of 0.3 ppm, the existing standard.

In April 1999, the U.S. EPA representatives met with a small group of Commodity Industry Coalition members, including R.J. Reynolds’s Seckar and Sciences International’s Anderson (Seckar 1999a). Anderson questioned the U.S. EPA’s interspecies uncertainty factor, citing several animal studies and an epidemiologic study to suggest that the U.S. EPA’s calculations were too conservative (Seckar 1999a). In an e-mail, Seckar noted that Anderson’s presentation was very effective, as evidenced by the fact that U.S. EPA representatives were now informing coalition members that the 0.03 ppm standard “was not ‘set in stone,’” a direct contradiction of earlier statements to the U.S. Department of Agriculture (Bair 1999; Seckar 1999d). (Despite Freedom of Information Act requests, we were unable to obtain U.S. EPA documents related to its meetings with the coalition.)

Soon after, Sciences International asked the Commodity Industry Coalition for additional funding to turn its phosphine report into a peer-reviewed journal article (Turim 1999). In a memo to Seckar, Anderson (1999b) explained that

My experience is that consultant reports funded by those being regulated, and written expressly for the EPA, are easily and frequently ignored or dismissed by the Agency, no matter how scholarly. However, a paper or article that is peer-reviewed and published, or in the peer review process for publication, in an accepted scientific journal can neither be ignored nor dismissed.

Anderson suggested that since she was editor-in-chief of *Risk Analysis*, “perhaps the peer review process could be expedited if we decide that it is the journal of choice” (Anderson 1999b). R.J. Reynolds, Brown and Williamson, and several other tobacco companies agreed to fund most of the cost of this work (Seckar 1999e). The paper was published in *Risk Analysis* in 2004, with the acknowledgment that “This work was supported by the Phosphine/Metal Phosphide Coalition, consisting of the producers and users of phosphine and metal phosphides for the control of insects in stored commodities” (Pepelko et al. 2004).

Coalition members also pursued other strategies. At a meeting with U.S. EPA representatives in March 1999, the Commodity Industry Coalition proposed that the U.S. EPA participate in a series of small, coalition-sponsored focus groups to “educate [EPA] on the issues involved with … fumigations” (Seckar 1999g). One such group met in May 1999, when tobacco companies demonstrated a tobacco warehouse fumigation (Ward and Cowan 1999). The following month, several companies conducted additional emissions tests to show that the proposed 500-foot buffer was unnecessary (Bridges 1995). However, an e-mail message from a Philip Morris employee indicated that Philip Morris’s test coordinator had “some reservations regarding the quality of the test design/data generation” and that he himself believed that “the test plan and methods will provide, literally, no information, so it won’t hurt us to do it” (Bridges 1995).

In June 1999, Sciences International submitted a first draft of its phosphine toxicity review to some coalition members (Sciences International 1999a). A reviewer from the coalition’s lobbying firm pointed out that the animal studies cited did little to support the idea that the interspecies uncertainty factor should be eliminated “since most [of the animals] appear to be rat or mouse strains with similar breathing characteristics” (Wilkinson 1999). Instead, the studies cited by Sciences International seemed to support the idea that phosphine called for a conservative standard, as they indicated that “phosphine is a very toxic material to most species tested” (Wilkinson 1999). Another reviewer noted that the uncertain and tentative tone of the report “will trigger concerns by EPA and they will say ‘if [an] expert in the field states that there remains great uncertainty, maybe we are on solid ground by being very conservative’” (Barolo 1999a). Sciences International staff revised the report, removing tentative statements and asserting that their work to date supported reducing the interspecies uncertainty factor to 1 (effectively eliminating it), thus preserving the existing exposure standard of 0.3 ppm (Sciences International 1999b). They submitted this revised interim report to the U.S. EPA in July 1999 (Sciences International 1999b). At a Commodity Industry Coalition meeting that same month, coalition consultant Dan Barolo, former director of the U.S. EPA’s Office of Pesticide Programs (OPP), reportedly urged members to speed their efforts because

phosphine is quite hazardous when used improperly. The more the Coalition slows the process, the greater the chance for an accident with possible fatalities, which would send EPA back into conservative mode and make it far more difficult for them to publish reasonable RMMs. (Seckar 1999f)

In August, John Whalan, a toxicologist at the U.S. EPA’s Health Effects Division, summarized in a memo his analysis of Sciences International’s interim report (Whalan 1999). He noted that

there is no precedent for using an [interspecies uncertainty factor] of 1 when establishing … an inhalation regulatory value in the Health Effects Division. The only time an interspecies [uncertainty factor] is not applicable is when human data are used. The available data do not support deviating from Agency policy, and the Coalition did not provide any new data. (Whalan 1999)

He also pointed out that Sciences International’s review of animal studies, intended to show that phosphine toxicity was relatively constant across species, was largely “irrelevant” because it did not include a comparison of toxicity for a small versus large mammal.

In September 1999, phosphine registrants and several coalition members again met with U.S. EPA officials to discuss alternative RMMs proposed by the coalition (Seckar 1999i). Instead of a 500-foot buffer and a 750-foot neighbor notification requirement, the coalition recommended a “site management plan” that required companies to develop emergency preparedness measures. The U.S. EPA asked the Commodity Industry Coalition to reword its proposals to specify how and when workers and bystanders would be informed of danger (Seckar 1999i). On the exposure limit for workers, the U.S. EPA now proposed a 0.1-ppm standard (reflecting a reduction from 10 to 3 in the interspecies uncertainty factor) based upon Sciences International’s interim report (despite the weaknesses noted by Whalan) (Seckar 1999i). (The U.S. EPA failed to provide memos or notes regarding this decision.)

In several fall 1999 memos to Seckar, Sciences International staff explained that they thought it would be difficult to convince the U.S. EPA to drop the interspecies uncertainty factor without human exposure studies (Anderson 1999a; Gray 1999). Commodity Industry Coalition members expressed reluctance to commit to human studies without confirmation that this would convince the U.S. EPA to “give up” the uncertainty factor (Barolo 1999b). Barolo commented to Seckar, “I do not believe it will be easy for OPP to abandon both safety factors. There are too many unknowns from children to endocrine to reliability of studies to absence of dog/monkey study. … Some day they are going to figure out there is a 0.1 ppm standard in other countries and the door will close” (Barolo 1999c).

Although Sciences International had not yet submitted to the U.S. EPA its full report on phosphine, in December 1999, the U.S. EPA made its final decision (Sharp 1999). (This decision was published in the *Federal Register* in February 2001 [U.S. EPA 2001]). The U.S. EPA now mandated a “fumigation management plan” like that proposed by the Commodity Industry Coalition (U.S. EPA 2000). The agency also eliminated the inter-species safety factor and left the old 0.3-ppm standard in place, on condition that phosphine registrants conduct additional research if Sciences International’s review was found to be inadequate (U.S. EPA 2000). A coalition member noted that “it is important to point out that this additional work will take years and that the current 0.3 ppm threshold will stay in place during that time” (Sharp 1999). R.J. Reynolds credited its leadership on the scientific issues with saving the company “many millions of dollars” (R.J. Reynolds 2000).

## Conclusion

Although others have charged that agencies responsible for protecting human health and the environment are unduly influenced by the industries they regulate (Abraham 2002; Huff 2002), it is rare to be able to study this process from the perspective of the regulated industry. This study provides documentation of the behind-the-scenes activities of an industry as it attempts to influence the regulatory process on matters that have a direct bearing on public health.

Our analysis has limitations. Given the sheer volume and limited indexing of the documents, it is impossible to ensure that we located all potentially relevant documents. Some may have been destroyed or concealed by the tobacco companies (Liberman 2002); others may have never been obtained in the legal discovery process. In addition, we had no access to pesticide company documents, except those in the tobacco documents archives. Finally, despite properly filed Freedom of Information Act requests, we were unable to obtain from the U.S. EPA documentation of its meetings with the industry’s Commodity Industry Coalition. All minutes of meetings with stakeholders should be part of the public record.

Despite these limitations, the case studies discussed here provide insight into tactics that the tobacco industry applies to a regulatory agency when trying to influence the outcome of a decision. These tactics go significantly beyond the usual approaches—such as participation in public comment periods and public meetings—to influence scientific and regulatory decision making. Tobacco industry tactics described in these cases include:

- Encouraging a chemical company (Zoecon) to advocate for high MRLs without any supporting data and directing that same company to gather information about international regulatory efforts on methoprene in a manner designed to hide the interest of the tobacco industry in this chemical;
- Attempting to forestall regulatory efforts on tobacco pesticides in the European Community by creating voluntary industry MRLs for a subset of chemicals;
- Hiring an ex-WHO scientist to participate (without disclosing his funding source) in the WHO regulatory effort on EBDCs;
- Hiring several ex-U.S. EPA scientists to influence the U.S. EPA’s regulatory decision making on phosphine;
- Hiring scientific consultants with instructions to marshal data to support the tobacco industry’s *a priori* arguments and funding consultants to publish a report supporting these arguments in a journal over which the consultants had influence;
- Staging fumigations for the U.S. EPA with the knowledge that the methodology was flawed and the results would show no emissions problem.

Yet, as the case of European MRLs showed, the tobacco industry does not always work together effectively to influence regulations. Tobacco companies may disagree about regulatory strategies or conclude that inaction is preferable to action that might have unintended consequences. Moreover, the fact that even voluntary, industry-friendly pesticide guidelines posed significant problems for Philip Morris underscores tobacco industry motivation for resisting or influencing more stringent, government-imposed regulations.

This study also raises questions about industry influence over regulatory agencies. In the case of WHO deliberations on EBDCs, the tobacco industry coordinated covert actions, hiding the financial ties and involvement of CORESTA. Rigorous disclosure requirements and oversight might have allowed the WHO’s agencies to judge more accurately the potential for bias related to conflicts of interest. In the case of the U.S. EPA’s review of phosphine, a regulatory agency appears to have been quite willing to cooperate with the industry and its consultants. This is a reminder of why regulatory processes were designed to be transparent and open to the public, and why “closed-door” meetings between regulators and industry have been ruled illegal (Federal Advisory Committee Act 1972; Registration Standards 2004; Special Review Procedures 2002).

Protection of the public interest hinges on an open process and regulatory agencies’ willingness to stand up to pressure from regulated industries. When these are in doubt, public confidence in the fairness and efficacy of regulations may be unwarranted. The resource disparities between powerful industries and public health organizations may also make it difficult to ensure that the public interest is fairly represented, particularly when discussions occur behind closed doors, as apparently occurred at the U.S. EPA. Increased public and media scrutiny of these processes could help ensure that public health considerations are weighed at least as heavily as commercial ones.

Finally, given the deadly epidemic of tobacco-caused disease, which kills an estimated 5 million people annually worldwide (WHO 2004), is it in the public interest for regulatory agencies today to continue facilitating standards that make it easier and less costly to grow, transport, store, and manufacture tobacco products?

## Figures and Tables

**Table 1:** Number of documents yielded by searches of tobacco company collections at the Legacy Tobacco Documents Library using selected key words and wildcards(*).

	Tobacco company				
Key word	American Tobacco	Brown and Williamson	Lorillard	Philip Morris	R.J. Reynolds
Pesticide(*)	224	232	872	7,632	6,095
Crop protection agent(*)	3	60	66	1,533	193
Kabat/methoprene	65	182	604	5,416	2,336
Dithiocarbamate/EBDC(*)	1	22	130	278	275
Phosphine	28	21	195	247	580
World Health Organization/WHO	909	2,047	6,769	28,902	14,024
Environmental Protection Agency/EPA	1,423	2,082	23,791	155,094	24,961
Agrochemical Advisory Committee	0	37	48	684	383

**Table 2:** Overview of case studies.

Pesticide	Regulatory action	Dates	Agency	Tactics	Outcome
Methoprene	MRL of 1.0 ppm	1991–1995	Malaysian pesticide board	Work through chemical industry partners to avoid raising tobacco issues, request higher MRL with no supporting research	MRL raised to 15 ppm
Methoprene, others	Industry concern about future MRLs	1991–1993	European Community	Attempt to create voluntary MRLs to forestall regulation	No voluntary MRLs, no EC regulations
EBDCs/ETU	Potential imposition of residue tolerances	1989–1993	UN FAO/WHO JMPR	Hire ex-WHO scientist to review EBDCs and ETU, covertly lobby and assist JMPR	ETU listed as not genotoxic, higher ADI assigned
Phosphine	15 proposed risk mitigation measures including worker exposure standard of 0.03 ppm	1998–2001	U.S. EPA	Hire consultant with EPA ties to challenge scientific basis of proposed exposure standard, write journal article	Worker exposure standard increased to 0.3 ppm

UN FAO, United Nations Food and Agricultural Organization.
